# Understanding Aboriginal Peoples’ Cultural and Family Connections Can Help Inform the Development of Culturally Appropriate Cancer Survivorship Models of Care

**DOI:** 10.1200/JGO.19.00109

**Published:** 2020-01-13

**Authors:** Eli Ristevski, Sharyn Thompson, Sharon Kingaby, Claire Nightingale, Mahesh Iddawela

**Affiliations:** ^1^ Monash University, Warragul, Victoria, Australia; ^2^ Latrobe Community Health Service, Traralgon, Victoria, Australia; ^3^ Monash University, Bendigo, Victoria, Australia; ^4^ Latrobe Regional Hospital, Traralgon, Victoria, Australia

## Abstract

**PURPOSE:**

To explore the cancer diagnosis, treatment, and survivorship experiences of Aboriginal people in the Gippsland region, Victoria, Australia, and identify factors critical to the development of a culturally appropriate cancer survivorship model of care.

**PATIENTS AND METHODS:**

Yarning circles were used to capture the stories of 15 people diagnosed with cancer and/or those of family members. Yarning circles were conducted in two locations in the Gippsland region. Sessions were facilitated by an Aboriginal Elder, audio recorded, and transcribed verbatim. Thematic analysis of the data were triangulated among three researchers and incorporated researcher reflexivity.

**RESULTS:**

Cultural connections and family were critical supports on the cancer journey. Putting the needs of the family first and caring for sick family members were more important than an individual’s own health. There was “no time to grieve” for one’s own cancer diagnosis and look after oneself. Cancer was a private experience; however, the constancy of deaths highlighted the importance of raising family awareness. Health professionals did not always understand the importance of people’s cultural and family supports in their treatment and recovery. There were negatives attitudes in hospitals when family come to visit, seeing family as too large and overstaying visiting times. Health professionals did not seek family assistance with communication of information to family members whose literacy level was low, nor did they include family in treatment decision-making. Access to services depended on family support with transport, finances, and family responsibilities, often resulting in lapses in treatment and follow-up services.

**CONCLUSION:**

Understanding the importance of Aboriginal peoples’ cultural and family connections can help to inform the development of culturally safe cancer survivorship models of care.

## INTRODUCTION

The disparity in cancer outcomes for Australian Indigenous people is widening.^1-4^ Between 1998 and 2016, the cancer mortality rate for Indigenous people rose by 23%, while the rate significantly declined for non-Indigenous Australians.^5^ Commonly attributed factors such as poorer access and use of screening programs, delays in diagnosis, lower rates of treatment, higher comorbidity rates, and socioeconomic status are only part of the explanation.^6,7^ For Indigenous people in rural areas, geographical barriers and distance to available treatment, coupled with higher financial expenses and time away from family and Country, affect cancer outcomes and survivorship.^8-11^^,^ The farther Indigenous people with cancer live from urban centers, the less likely they are to survive their disease.^4^

There are significant gaps in the provision of culturally appropriate services to engage Indigenous people, and in the understanding of Indigenous Australians’ perspectives of health and illness and social and cultural determinates of health. Communication strategies are ineffective between health professionals and patients and their families.^12-15^ Aboriginal Hospital Liaison Officers (AHLOs) are one part of a strategy to assist Indigenous patients and their families navigate the health system and to promote culturally appropriate practices to all staff in the health system.^16,17^

**CONTEXT**

**Key Objective**
To determine factors critical to the development of cancer survivorship models of care for Australian Indigenous people.
**Knowledge Generated**
Culture and family were central themes and need to be at the core of cancer survivorship models of care. Culture affects health seeking, treatment decision-making, and acceptance of follow-up care. Family is important for psychosocial support in and out of hospital, decision-making, communication with health professionals, financial support, and access to health services.
**Relevance**
Cancer survivorship models of care need a greater focus on social and cultural determinates of health and practical strategies to support patients and families in follow-up and self-management. Models should include cultural supports as part of the multidisciplinary care team and should move beyond the hospital setting, building information and communication processes with mainstream and Aboriginal community health and primary care organizations to support cancer survivors in community settings.

Acknowledgment and respect for Indigenous family structures^18-20^ are an important area for service improvement that will better enable the key role that family and community play in improving survivorship outcomes. Family provides connection to identity, culture, spirituality, community, and Country. Recognition of this has been proposed as one of the four key pillars of a well-being framework for Aboriginal people with chronic illness^21^ and in Optimal Care Pathways for Aboriginal and Torres Strait Islander people with cancer in Australia.^22^ Mainstream health systems often fail to use the strength that Indigenous people gain from their community and from having a supportive family network.^23-25^ In addition, Indigenous people report drawing strength from the support that they provide to others within their community and how this positively affects their survivorship.^18^ Finally, family, community, health, and wellness are embedded within Indigenous people’s connection to Country, which ties people to their ancestors. This includes spiritual connections to place and the keeping and passing of knowledge and responsibilities. Identity, language, kinship, and culture are defined by connections to Country.^26^

The Gippsland Cancer Survivorship Program (GCSP) supports the transition of patients with cancer from active treatment to the post-treatment phase (ie, survivorship care). Gippsland is a rural location in eastern Victoria, Australia, with a population of 145,000 people geographically distributed over 42,000 km^2^. Cancer survivorship rates in Gippsland are the lowest in Victoria, with a markedly high incidence of asbestos-related disease. Household incomes are significantly below the Victoria state average.^27^ The GCSP uses a shared-care model among hospital medical oncologists, oncology nurses, general practitioners, and patients in three hospitals across Gippsland, with one hospital offering teleconferencing consultations. A cancer Survivorship Care Plan (SCP) is developed with the cancer survivorship nurse. SCPs include treatment history, follow-up care schedule, supportive care and quality-of-life assessment, and personal health and well-being goals. Although shared-care models are effective in non-Indigenous people with cancer,^28-30^ there are no published data, to our knowledge, regarding their use with Indigenous patients with cancer. In this study, we explored the cancer diagnosis, treatment, and survivorship experiences of Indigenous people in Gippsland to identify factors critical to the development of a culturally appropriate cancer survivorship model of care.

## METHODS

### Recruitment

This research was conducted in the Gippsland region of Victoria, Australia. People in Gippsland identify as Aboriginal and we respectfully use this term in the following discussion. Flyers were distributed by staff at Aboriginal community organizations and health services, Elders groups, and primarily through word of mouth by the community and respected Aboriginal people. Aboriginal people diagnosed with cancer and their families were invited to share their stories. Information and consent forms and/or verbal explanations of the research aims and processes were provided. Ethical approval for the research was obtained through the Latrobe Regional Hospital Human Research Ethics Committee (no. 2017-01LNR).

### Participants

A total of 15 people participated in the study; four men and 11 women. Participants were between 30 and 70 years of age. Three had undergone cancer treatment and others shared stories about partners or family members diagnosed with cancer. On average, each participant shared stories pertaining to three family members affected by cancer.

### Data Collection: Yarning Circles

Yarning, a culturally and methodologically relevant approach to sharing information, knowledge, and culture, was used to collect data. Although there are numerous methods of conducting yarning circles, yarning processes must ensure inclusivity and respect for each person’s views.^31-33^ The two yarning circles in this study were different in process to reflect the dynamics of the participants in the group. One yarning session used a “talking stick,” which when held, allowed that person to tell their story without interruption or comment. The other yarning circle was primarily a focus-group discussion during which participants raised topics and shared experiences. In each circle, Elders were invited to start the discussion. The yarning circles were facilitated by an Aboriginal Elder known and respected by the community, held in different locations in community facilities, were conducted over 2 hours, were audio recorded and transcribed verbatim. The experience of the facilitator and connection to community created a culturally safe space for participants to share information.^34^ The facilitator also shared experiences illustrating experiential knowledge, trust, and reciprocity in the sharing of personal stories. Topics for discussion were used rather than a formal interview schedule and included feeling unwell, diagnosis, getting treatment, and treatment experiences.

### Thematic Analysis

Yarning sessions were analyzed with thematic analysis, triangulated among three researchers, and incorporated researcher reflexivity.^35^ Reflexivity involved (1) reflecting on the research and data collection process (factors that may have influenced participants’ responses) and (2) the interpretation of the data (ie, what personal experiences, knowledge, values, and bias does the researcher bring to the analysis?). The triangulation of expertise of the three researchers, which included oncology research, qualitative methodology, social and cultural determinates of health, work in community and Aboriginal Health Services and Aboriginality, increased the authenticity of the analysis. Thematic analysis is an inductive process of identifying common experiential themes, topics, meanings, and patterns in the data.^36^ Each researcher independently reviewed the transcripts and grouped the participants’ words and phrases to form concepts. The concepts were named, using either the participants’ own words or words the researcher had identified in the research literature. The three researchers then worked together to collapse, expand, and create new concepts, forming provisional themes. Themes were united concepts and included additional theoretical interpretation of the data that were additionally analyzed to identify relationships, make comparisons, and note contrasting or emerging themes. This process formed the final themes and was complete when there was consensus by the three researchers that the analysis had reached saturation; that is, no new themes emerged.

## RESULTS

Seven key themes emerged (Table 1): culture, family, cancer is private, health services and staff, communication and information, hospital cultural supports, and access.

**TABLE 1:**
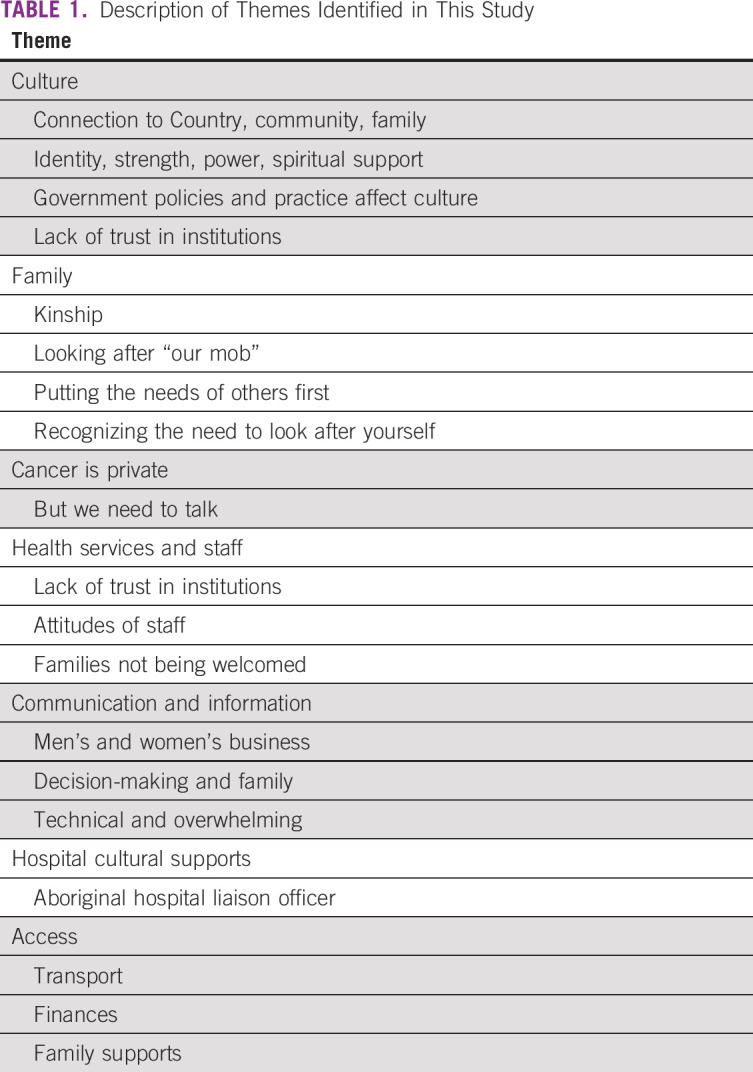
Description of Themes Identified in This Study

### Culture

Culture underpinned all the themes and influenced people’s actions and responses. Culture related to connection with Country, community, and family. Culture shaped identity, gave strength and power to heal, and provided spiritual support.

“Our culture has lifted me from where I was, ‘cause I was down here, now I’m back up here, and I give thanks to all the old fellas ‘cause they’ve given me strength in places I never thought even existed” (FG1, female).

“A spiritual side of that I think has brought us a lot of comfort. And that really healed some really gaping wounds in the kids too … So we talk about him like he’s still around, he’s still a part of our decision-making” (FG1, female).

However, culture has been severely affected by past and current government practices. The Stolen Generations, referring to when Aboriginal children were forcibly removed from their families and denied their culture, caused significant personal, social, and community angst. A lack of trust in institutions underpins and shapes Aboriginal peoples’ trust in current practices:

“If it’s got this big tag of the Government on it. I won’t go anywhere near it. As Aboriginal people, we’ve been scrutinized and chucked from pillar to post by the Government. So we start griping when we get anything from such a bureaucracy” (FG1, male).

### Family

Unlike western cultures, Aboriginal family is defined as a complex system of kinship structures, roles and responsibilities, and connection to culture and Country. Family includes people who are not necessarily genetically related, was described in the yarning circles as a “spider’s web of connections” between people and generations. The following comment describes the cultural and kinship connection and connection to family from 40,000 years ago:

“Remember how I said that your nanna and my dad, we go way back, and there's an old man, and he was 40,000 years old, and you and me, that’s our relative, that’s our old poppie, you could say great, great, great, great, great, great for an hour, and that’s our pop, and we’re related to that old man, you and me” (FG1, male).

Looking after “our mob” (ie, family and community) is part of Aboriginal people’s roles and responsibilities. With the passing of family members, new responsibilities emerge: “As soon as someone goes. you have this new sense of responsibility. You go through all this grieving, but then you’ve also got to put these big shoes on to take responsibility” (FG1, male). Even after people had separated from relationships, they still provided care for former partners and family: “They’d been separated for years but she went back and looked after him for his last couple of years. She slept in a little bed beside his bed” (FG2, female).

Putting the needs of others first and caring for sick family members came before an individual’s own health or priorities. There was “no time to grieve” for their own diagnosis and look after themselves: “We go through these tragedies, whether they be health issues or, and they may not necessarily go through them ourselves, we still have to deal with it” (FG1, female).

There was recognition if one does not look after oneself, one could not look after others: “At one stage, I had 15 children in my house, always putting them first before myself. If I don’t start looking after myself, god forbid, if anything happened to me, who would do that?” (FG1, female).

### Cancer is Private, But We Need to Talk

Cancer is seen as private and “not spoken about.” “It’s just the way we are, we don’t talk about them things, ‘cause as soon as you start talking about it, it festers all this juju, bad juju” (FG1, female). Words such as “shocking disease,” “evil thing,” and “I hate cancer” were used. Watching family and community die of cancer prompted the need to talk about it. Although cancer is seen as a private issue, people described a need for more awareness among family and community members to encourage participation in screening and health checks to save lives and dispel the myths about screening: “It takes our mob, and like our mob doesn’t get checked up on it, or it’s embarrassing, or their life takes over” (FG2, female).

### Health Services and Staff

The lack of trust in institutions was also present in people’s experiences with health services, and hospitals were usually associated with death and dying, not recovery. When a person was unwell, being “on Country” was important to where they received treatment and how it aided in their recovery.

In hospitals, mainstream health professionals did not appreciate the importance of people’s cultural and family supports in their treatment and recovery. When family came to visit, there were negative attitudes from staff who saw family as too large and overstaying visiting times.

“The family is rallying around you to show their support, and they’re told ‘listen, too many of you here.’ That’s not the black fella way to be told too many family here. Well that’s what we’re here for, is our family” (FG1, female).

Having family present reduced feelings of isolation and increased feelings of safety: “I felt more comfort when all the family turned up …there was just a feeling of being safe … and comforted” (FG1, female). Patients carried the emotional burden of their families not being welcomed into health services and felt they had no voice to explain the personal and cultural importance of family in their treatment and recovery. People talked about self-discharging from hospital because they did not feel culturally safe.

### Communication and Information

Inclusive communication between staff and patients and their families was seen as problematic. Conversations about treatment should respect cultural protocols of where, and with whom, it is appropriate to talk, especially considering “men’s or women’s business.” Health professionals did not seek family assistance to communicate information to patients who had literacy difficulties, nor did they include family in treatment decision-making. Participants described family members signing documents when they could not read, without being offered support to understand those documents.

The language used to communicate information was too technical and overwhelming, and there was missed information about treatment and medication adverse effects after returning home. Written information was the most common mode of communication and information sharing. People who could not read felt overwhelmed by their diagnosis or treatment process, and they missed vital information, making family involvement extremely important. “Communication between nurses and doctors and community can get very skewed, ‘cause they talk in a different language, they need to keep it simple” (FG1, female).

### Hospital Cultural Supports

The involvement of an AHLO in the hospital was identified as a culturally safe source of support for patients and families. Although many health services use AHLOs, referrals had not occurred. An AHLO with knowledge and understanding of cancer and treatments could provide information, cultural supports, and be an advocate for the individual and their family. This would help the health service understand the importance of cultural and personal circumstances and remove pressure from families to be the sole advocate and voice for the patient. The opportunity to talk with someone other than a family member was important for patients because they felt family carried much of the emotional burden.

“Having a black fella there to support you, just even a nurse or someone from the Co-Op [Aboriginal Health Service] to come down and say you know ‘it’s going to be okay.’” (FG2, female).

### Access

Access to services, including treatment and follow-up, depended on family support for transport, finances, and managing responsibilities while away from home. Transport to medical appointments, locally or in the metropolitan area, continually arose as an issue and the distance to services was compounded by people not driving, or not having a driver’s license, a car, or the money to buy petrol. Also, access to public transport in rural areas was limited, with the added burden of traveling while unwell and not having the money to pay the fare.

“First train goes out of here at 6:20 am and it gets to [city] at 10 am something, hospitals are on the other side of the [city]. To get back here, you’ve got to get on the 5 o’clock train in the afternoon and arrive home at 10 o’clock at night” (FG1, female).

Patient-transport subsidy schemes were paid retrospectively and were based on the assumption that people had the money up front. People relied on family for transport to appointments, and often family had to stay overnight. “Be mindful that you’ll be fairly crook, so they get a ride down, but then the family’s got to stay in town for weeks on end maybe” (FG1, female).

Costs for hospital parking, overnight accommodation, and meals left families with significant out of pocket expenses:

“You’ve got to get home; you’ve got accommodation, if you don’t have family or anything nearby, you’ve got to pay for that. The hospital accommodation, you’ve still got to pay for it. I know there’s a government subsidy program that you can get some of your money back, but it takes so long.” (FG1, female).

Family can also feel obligated to provide financial support to those in hospital, and it was not unusual to ask to borrow money, assuming the family had money to spare: “You can’t be loaning family money when you haven’t got any yourself, you know, you’d flick ‘em 50 bucks to get petrol, but you can’t.” (FG1, female).

## DISCUSSION

We found that culture and family were central to treatment and survivorship experiences of Aboriginal patients with cancer and must be at the center of cancer survivorship models of care to positively affect cancer outcomes for Aboriginal patients. When culture is strong, Aboriginal people’s well-being and health outcomes are improved.^5,37,38^ Culture affects health seeking, treatment decision-making, and acceptance of follow-up care.^39,40^ We found that culture and family connections provided strong personal and spiritual support during the cancer journey, improving cancer survivorship outcomes. Tam et al^24^ found support from family and friends was the most frequently mentioned facilitating factor in improving the resilience and optimism of Aboriginal patients during cancer treatment and recovery. In a systematic review by Cavanagh et al,^25^ family presence and involvement were important in enabling follow-up care.

In our study, we found that although family filled a critical role during treatment and into survivorship, putting the patient and other family members first was at the expense of their own health. This finding is not uncommon for people in caring roles.^41^ For Aboriginal people, cultural roles and responsibilities add another dimension to the care role they undertake.^42,43^ The study showed that Aboriginal families felt they did not receive supportive care and did not feel welcome in the hospital. The National Aboriginal and Torres Strait Islander Cancer Framework^44^ states that families and carers need to be “involved, informed, supported and enabled throughout the cancer experience.”^44^ This Framework, used in conjunction with the National Safety and Quality Health Service Standards,^45^ can assist in the review and redesign of service delivery that includes families and reports progress across this standard of care.

This research also highlights the far-reaching financial impact of cancer on Aboriginal patients and their families. As previously discussed, there was reliance on family for financial support while having treatment away from home. Although there is much discussion about financial toxicity for patients with cancer,^46-48^ the true costs and impacts for Aboriginal families are significantly higher than for non-Aboriginal populations.^49^ For Aboriginal people in rural areas, distance adds another social and economic hardship to the cancer treatment and survivorship experience.^25,50,51^ GCSP uses telehealth at one site to develop an SCP between the patient and the nurse. Early findings show high patient satisfaction with the service, but there is still preference for a face-to-face consultation with the nurse, suggesting need for additional exploration of this model.

Discussing cancer with family and raising community awareness for screening were also key findings from this research. The word cancer does not exist in many Indigenous languages^52^ and has been associated with a death sentence. This fear, along with the minimal presence of Aboriginal people in health promotion and media campaigns, creates a silence in private and public discussions about cancer.^53^ In our study, the continual deaths in families and the missing information on family medical histories highlighted the increased benefits of discussing cancer. Cancer survivorship shared-care models with primary health providers, community health services, and Aboriginal community–controlled organizations can provide safe places for Aboriginal people and families to access information about health care services and support.

At the health service level, particularly hospitals, the importance of culture in the treatment and recovery of Aboriginal patients was not understood. Families felt unwelcome in the health service and by the treatment team. The positive and critical role that family carers play in cancer recovery is widely recognized and part of best practice,^37^ yet the inclusion of families appears not to occur for Aboriginal people. Many health services and professionals applied western definitions of family, not understanding Aboriginal family kinship structures were culturally relevant to Aboriginal people. Our study supports the need for further work into developing culturally safe services. There has been a move at the national policy level in Australia to implement change in this area. The Australian Commission on Safety and Quality in Health Care^45^ requires health organizations to address six action areas specific to Aboriginal and Torres Strait Islander people that demonstrate welcoming environments, that recognize the importance of cultural beliefs and practices and improve cultural awareness and competency in the workforce and partnering with consumers to meet their health care needs. Similar to other authors, we argue that cultural awareness training is not enough; it should be part of a suite of initiatives to increase collaboration among health services, practitioners, and Aboriginal people to instill culturally safe practices.^54-56^ Furthermore, cultural safety initiatives driven at the senior management level have greater impact across the whole organization.^45,57^

Our study also found gaps in information sharing and communication between health professionals and patients and their families. Most concerning was Aboriginal patients with low literacy levels who were asked to sign medical documentation they could not read and/or understand. Family can be instrumental in providing support to the patient and the health care team. Providing information in plain language facilitates informed decision-making, understanding of diagnosis, treatment, and follow-up care.^24,58,59^ A systematic review of culturally safe health care communication by Jennings et al^60^ found when health professionals communicated with patients and families, a greater sense of care and humility was created, and this influenced adherence to treatment plans and continued use of the health service. Furthermore, the use of language and communication showing cultural respect reduced power differentials between patients and practitioners.^60^

Finally, our research highlights the importance of AHLOs in the cancer treatment trajectory. AHLOs were seen as providing cultural support, a conjugate between patients and staff, and as an advocate for the patient and their family. Many people stated they had not been referred to or had seen an AHLO during their treatment or transition to survivorship. Several studies have shown the benefits a culturally appropriate navigator has on the patient experience in the hospital setting.^61-63^ However, with a limited Aboriginal health workforce and the necessity to support people from multiple disease groups, AHLOs are already stretched.^64^ Even in large health services, there may only be one AHLO. The transition to survivorship requires connection back into community health services and general practice, a shared-care role that could be supported by Aboriginal community–controlled organizations and seen as appropriate for Aboriginal people.^65^ Although AHLOs are seemingly a good solution, funding would be required for Aboriginal services to provide this role, which would involve including an increased workforce capacity, and cancer and organizational systems and process education.^66-68^

Our study is limited by the small number of participants and of people diagnosed with cancer who participated. However, our findings provide new insight into the experiences of Aboriginal people in this part of Australia, illustrating the diversity of Aboriginal people and experiences in a geographically and culturally vast county such as Australia.

In conclusion, in this study, we identified the important role of culture and family in supporting Aboriginal people through their cancer journey and transition to survivorship. These findings can assist health services to develop and review cancer survivorship models of care. Models of shared care need to incorporate cultural supports as part of the team. SCPs should have greater focus on social and cultural determinates of health and practical strategies to support patients and families in follow-up and self-management. Cancer survivorship programs need to move beyond the hospital setting, building communication processes with mainstream and Aboriginal community health and primary care organizations to support cancer survivors in a community-based setting. Telehealth models in cancer survivorship need to be tested more in rural areas to assess if they improve access to cancer survivorship services.
